# Concerns over use of glyphosate-based herbicides and risks associated with exposures: a consensus statement

**DOI:** 10.1186/s12940-016-0117-0

**Published:** 2016-02-17

**Authors:** John Peterson Myers, Michael N. Antoniou, Bruce Blumberg, Lynn Carroll, Theo Colborn, Lorne G. Everett, Michael Hansen, Philip J. Landrigan, Bruce P. Lanphear, Robin Mesnage, Laura N. Vandenberg, Frederick S. vom Saal, Wade V. Welshons, Charles M. Benbrook

**Affiliations:** Environmental Health Sciences, Charlottesville, VA, and Adjunct Professor, Carnegie Mellon University, Pittsburg, PA USA; Department of Medical and Molecular Genetics, Faculty of Life Sciences and Medicine, King’s College London, London, UK; Department of Developmental and Cell Biology, University of California, Irvine, CA USA; The Endocrine Disruption Exchange, Paonia, CO USA; L. Everett & Associates, Santa Barbara, CA USA; Consumers Union, Yonkers, NY USA; Department of Preventive Medicine, Icahn School of Medicine at Mount Sinai, New York, NY USA; Child & Family Research Institute, BC Children’s Hospital, University of British Columbia, Vancouver, BC Canada; Department of Environmental Health Sciences, School of Public Health and Health Sciences, University of Massachusetts – Amherst, Amherst, MA USA; Division of Biological Sciences, University of Missouri, Columbia, MO USA; Department of Biomedical Sciences, University of Missouri-Columbia, Columbia, MO USA; Benbrook Consulting Services, 90063 Troy Road, Enterprise, OR 97828 USA; Environmental Health Sciences, 421 Park St, Charlottesville, VA 22902 USA

**Keywords:** Glyphosate, Acceptable daily intake (ADI), AMPA, Consensus statement, Endocrine disruptor, Reference dose (RfD), Risk assessment, Roundup Ready, Toxicology

## Abstract

The broad-spectrum herbicide glyphosate (common trade name “Roundup”) was first sold to farmers in 1974. Since the late 1970s, the volume of glyphosate-based herbicides (GBHs) applied has increased approximately 100-fold. Further increases in the volume applied are likely due to more and higher rates of application in response to the widespread emergence of glyphosate-resistant weeds and new, pre-harvest, dessicant use patterns. GBHs were developed to replace or reduce reliance on herbicides causing well-documented problems associated with drift and crop damage, slipping efficacy, and human health risks. Initial industry toxicity testing suggested that GBHs posed relatively low risks to non-target species, including mammals, leading regulatory authorities worldwide to set high acceptable exposure limits. To accommodate changes in GBH use patterns associated with genetically engineered, herbicide-tolerant crops, regulators have dramatically increased tolerance levels in maize, oilseed (soybeans and canola), and alfalfa crops and related livestock feeds. Animal and epidemiology studies published in the last decade, however, point to the need for a fresh look at glyphosate toxicity. Furthermore, the World Health Organization’s International Agency for Research on Cancer recently concluded that glyphosate is “probably carcinogenic to humans.” In response to changing GBH use patterns and advances in scientific understanding of their potential hazards, we have produced a Statement of Concern drawing on emerging science relevant to the safety of GBHs. Our Statement of Concern considers current published literature describing GBH uses, mechanisms of action, toxicity in laboratory animals, and epidemiological studies. It also examines the derivation of current human safety standards. We conclude that: (1) GBHs are the most heavily applied herbicide in the world and usage continues to rise; (2) Worldwide, GBHs often contaminate drinking water sources, precipitation, and air, especially in agricultural regions; (3) The half-life of glyphosate in water and soil is longer than previously recognized; (4) Glyphosate and its metabolites are widely present in the global soybean supply; (5) Human exposures to GBHs are rising; (6) Glyphosate is now authoritatively classified as a probable human carcinogen; (7) Regulatory estimates of tolerable daily intakes for glyphosate in the United States and European Union are based on outdated science. We offer a series of recommendations related to the need for new investments in epidemiological studies, biomonitoring, and toxicology studies that draw on the principles of endocrinology to determine whether the effects of GBHs are due to endocrine disrupting activities. We suggest that common commercial formulations of GBHs should be prioritized for inclusion in government-led toxicology testing programs such as the U.S. National Toxicology Program, as well as for biomonitoring as conducted by the U.S. Centers for Disease Control and Prevention.

## Background

This Statement of Concern is directed to scientists, physicians, and regulatory officials around the world. We highlight changes in the scope and magnitude of risks to humans and the environment stemming from applications of glyphosate-based herbicides (GBHs). The objectives of this statement are to: 1) demonstrate the need for better monitoring of GBH residues in water, food, and humans; (2) identify limitations or weaknesses in the way the EPA, the German Federal Institute for Risk Assessment, and others have previously assessed the potential risks to humans from exposure to GBHs; and (3) provide recommendations on data needs and ways to structure future studies addressing potential health risks arising from GBH exposures.

Our focus is on the unanticipated effects arising from the worldwide increase in use of GBHs, coupled with recent discoveries about the toxicity and human health risks stemming from use of GBHs. Our concern deepened when the World Health Organization’s International Agency for Research on Cancer (IARC) re-classified glyphosate as “probably carcinogenic to humans” (i.e., Group 2A) [1].

We highlight a number of issues that influence our concern about GBHs including: 1) increased use of GBHs over the past decade, including new uses for these herbicides just prior to harvest that can lead to high dietary exposures; 2) detection of glyphosate and its metabolites in foods; 3) recent studies that reveal possible endocrine system-mediated and developmental impacts of GBH exposures; and 4) additional complications for farmers, most acutely the emergence and spread of weeds resistant to glyphosate and the concomitant use of multiple herbicides in mixtures, both of which increase the risk of human and environmental harm. We discuss evidence pointing to the need to adjust downward the acceptable daily intake for glyphosate. Our major concerns are embodied in a series of consensus points that explicitly address the strength of the supporting evidence, and our recommendations focus on research essential in narrowing uncertainty in future GBH risk assessments.

When regulatory agencies conducted their initial assessments of glyphosate toxicity (in the 1970s) and approved a wide array of agricultural and non-agricultural uses, only limited and fragmentary data on GBH toxicity and risks were available. Testing done by contract laboratories were commissioned by the registrant and submitted to regulatory agencies. Results indicated minimal mammalian toxicity. A large review published in 2000, written by consultants associated with the registrant and drawing on unpublished industry reports, agreed with and reinforced that conclusion [2]. However, their review did not address some statistical differences reported between test and control groups that could be interpreted more cautiously, and surely warrant further assessment [3, 4].

In killing weeds and indeed almost all growing plants, the primary mode of glyphosate herbicidal activity is the inhibition of a key plant enzyme, namely 5-enolpyruvylshikimate-3-phosphate synthase (EPSPS). This enzyme is part of the shikimic acid pathway and is essential for the synthesis of aromatic amino acids that govern multiple, essential metabolic processes in plants, fungi, and some bacteria. Since this EPSPS-driven pathway does not exist in vertebrate cells, some scientists and most regulators assumed that glyphosate would pose minimal risks to mammals. However, several studies, some described below, now show that GBHs can adversely affect mammalian biology via multiple mechanisms.

### Glyphosate use is increasing significantly

The United States has the world’s most complete, publicly accessible dataset on GBH use trends over the past 40 years. Usage trends have been analyzed by EPA in a series of pesticide sales and use reports spanning 1982–2007 [5, 6], U.S. Geological Survey scientists [7, 8], the USDA’s National Agricultural Statistics Service (NASS) [9], and academic and industry analysts [10–12].

Briefly, glyphosate was registered in 1974 in the U.S. Initially, this broad-spectrum, contact herbicide was sprayed by farmers and ranchers primarily to kill weeds before the planting of fields, and for weed control in pastures and non-crop areas. In 1987 between 6 and 8 million pounds (~2.72–3.62 million kilograms) were applied by U.S. farmers and ranchers [5]. In 1996, the first year genetically engineered (GE), glyphosate-tolerant crops were planted commercially in the U.S., glyphosate accounted for just 3.8% of the total volume of herbicide active ingredients applied in agriculture [7].

By 2007, the EPA reports agricultural use of glyphosate in the range of 180–185 million pounds (~81.6–83.9 million kilograms) [6]. The USGS team projects that glyphosate accounted for 53.5% of total agricultural herbicide use in 2009 [7]. In the 20-year timespan covered by EPA sales and usage reports (1987–2007), glyphosate use rose faster and more substantially than any other pesticide. Usage in the range of 81.6–83.9 million kilograms, which occurred in 2007, was more than double the next most heavily sprayed pesticide (atrazine, 73–78 million pounds; ~33.1–35.4 million kilograms). For over a decade, GBHs have been, by far, the most heavily applied pesticides in the U.S.

By 2014, annual farm-sector glyphosate usage increased to approximately 240 million pounds (~108.8 million kilograms), based on average annual crop use reported by the NASS [9, 12]. Available use data published by the USDA, USGS, and EPA show that a surprisingly large share (approximately two-thirds) of the total volume of GBH applied since 1974 has been sprayed in just the last decade.

### Glyphosate residues are found in foods

GBHs are widely used on a range of crops including maize, soy grain, canola, wheat, barley, and edible beans, among others [9]. GBH application to these crops can result in residues of glyphosate and its primary metabolite AMPA in crops at harvest [13], as well as in processed foods. For example, the UK-Food Standard Agency residue testing conducted in October 2012 found glyphosate residues at or above 0.2 mg/kg in 27 out of 109 samples of bread [14]. Testing by the US Department of Agriculture in 2011 revealed residues of glyphosate in 90.3% of 300 soybean samples, and AMPA in 95.7% of samples at concentrations of 1.9 ppm and 2.3 ppm respectively [13]. Other laboratories have reported much higher levels in soybeans in recent years (e.g., [15, 16]).

Late season, harvest aid use of GBHs is an important new contributor to the increase in residue frequency and levels in some grain-based food products. This is particularly true in humid, temperate-climate countries such as the UK. Such applications are made within one to two weeks of harvest to accelerate crop drying, thus permitting harvest operations to begin sooner (a so-called “green burndown” use [17]). Such late season applications typically result in much higher residue levels in the final harvested product compared to crops subjected to typical application rates at earlier stages in the crop growth cycle. Pre-plant applications of GBHs, as well as post-harvest or fallow period applications, rarely result in detectable residues in grain, oilseeds, or forage crops.

### Data from humans and laboratory animals indicate hazards associated with exposure

Classical toxicity studies assess high doses and examine ‘validated’ endpoints – those that have been shown to be replicated easily in many laboratories [18]. Although these endpoints are known to represent adverse outcomes, they typically do not correlate with human diseases, and are not considered comprehensive for all toxicological endpoints [19, 20]. Regulatory long-term (2 year) toxicity studies in rodents revealed adverse effects of glyphosate on the liver and kidney (reviewed in [3, 4]). These studies, however, typically do not address a wide range of potential adverse effects triggered by disruption in endocrine-system mediated developmental or metabolic processes [3, 21–24]. Studies examining low doses of GBHs, in the range of what are now generally considered ‘safe’ for humans, show that these compounds can induce hepatorenal damage [25–28].

Concerns about the carcinogenic properties of GBHs have increased after the World Health Organization’s International Agency for Research on Cancer (IARC) re-classified glyphosate as “probably carcinogenic to humans” [1]. This decision was based on a small number of epidemiological studies following occupational exposures, rodent studies showing associations between glyphosate and renal tubule carcinoma, haemangiosarcoma, pancreatic islet cell adenoma, and/or skin tumors, and strong, diverse mechanistic data.

Human epidemiological [23, 29–31] and domesticated animal studies [32, 33] suggest associations between exposures to GBHs and adverse health outcomes. For example, congenital malformations have been reported in young pigs fed GBH residues-contaminated soybeans [32]. This suggests that GBHs may be at least a contributing factor to similar birth defects observed in human populations living in and near farming regions with substantial land area planted to GBH-tolerant GE crop cultivars [23, 34].

Collectively, studies from laboratory animals, human populations, and domesticated animals suggest that current levels of exposure to GBHs can induce adverse health outcomes. Many of these effects would likely not be detected in experiments adhering to traditional toxicology test guidelines promulgated by pesticide-regulatory authorities.

### Further complications: resistance and mixtures

Genetically engineered crops with tolerance to glyphosate are widely grown, and their use has led to increased application of GBHs [10, 35]. This increased use has contributed to widespread growth of glyphosate-resistant weeds [36, 37]. To combat the proliferation of glyphosate-resistant weeds, GE plant varieties have been approved for commercial use that are resistant to multiple herbicides, including several older compounds that are possibly more toxic and environmentally disruptive than GBHs (for example, 2,4-D and dicamba).

While farmers have struggled for 30 years with the steady increase in the number of weeds resistant to one or more herbicides, the geographic scope and severity of the weed control challenges posed, worldwide, by the emergence and spread of glyphosate-resistant weeds is unprecedented [37]. Moreover, the consequences triggered by the spread of glyphosate-resistant weeds, in contrast to the emergence in the past of other herbicide-resistant weeds, are unparalleled, and include the need for major changes in tillage and cropping patterns, and large increases in farmer costs and the diversity and volume of herbicides applied [10, 36, 38, 39].

In addition to resistance, concerns have been raised about the toxicity of herbicide mixtures, because current data suggest that chemicals in combination can have effects that are not predicted from tests of single compounds [40, 41]. GBHs themselves are chemical mixtures; in addition to the inclusion of glyphosate (the active ingredient), these herbicides include adjuvants such as surfactants, which can make GBH-product formulations more toxic than glyphosate alone [42–44]. In light of the increased numbers, levels and extent of herbicide use elicited by weed resistance, it is reasonable to predict that there will be a marked increase in the diversity of biological pathways affected, the number and duration of high-exposure periods, and the magnitude of potential risks facing non-target organisms, including humans. Such impacts could be limited, or even largely prevented, if there are substantial changes in weed-management systems and regulatory policy, including enforceable limits on herbicide-use patterns known to cause relatively high and potentially unsafe residue levels in food, water, and the air.

### Setting an acceptable intake level of GBHs

Different countries have established a range of “acceptable” daily intake levels of glyphosate-herbicide exposures for humans, generally referred to in the U.S. as the chronic Reference Dose (cRfD), or in the E.U. as the Acceptable Daily Intake (ADI).

The current U.S. Environmental Protection Agency (EPA) cRfD is 1.75 mg of glyphosate per kilogram body weight per day (mg/kg/day). In contrast, the current E.U. ADI is more than 5-fold lower at 0.3 mg/kg/day, a level adopted in 2002. The data upon which these exposure thresholds are based were supplied by manufacturers during the registration process, are considered proprietary, and are typically not available for independent review.

The German Federal Institute for Risk Assessment is the lead regulatory authority currently conducting an E.U.-wide reassessment of GBHs. Their renewal assessment report calls for an increase of the E.U. ADI from 0.3 mg/kg/day to 0.5 mg/kg/day [45]. However, from an analysis of their assessment, it is difficult to understand the basis on which the German regulators are making this recommendation, since they still rely on the same proprietary, industry-supplied dataset that led to setting a lower ADI (0.3 mg/kg/day) in 2002. In contrast, an international team of independent scientists concluded that the current E.U. ADI is probably at least three-fold too high, based on a transparent, fully documented review of the same dataset [3]^1^.

In December 2009, the U.S. EPA’s re-registration review of glyphosate identified a number of issues of ongoing concern, as well as GBH data gaps [46]. In particular, it noted that data relating to the effects of GBHs on the immune and neurological systems were limited and announced that future registrants would be required to conduct both neurotoxicity and immunotoxicity studies. The U.S. EPA’s updated risk assessment and final re-registration decision on GBHs is scheduled to be completed in 2015–2016.

As noted above, most GBH use has occurred in the last 10 years, while most studies considered by regulatory agencies for the assessment of GBHs focused just on the active ingredient, and were conducted in the 1970s through mid-1980s. Since the late 1980s, only a few studies relevant to identifying and quantifying human health risks have been submitted to the U.S. EPA and incorporated in the agency’s GBH human-health risk assessment^2^.We believe that the ability to establish appropriate GBH exposure and use levels should be enhanced and grounded in “up-to-date science” to support refined and accurate assessments of GBH health risks and to assure that regulators understand both the likely and possible consequences of the decisions they make.

Table 1 lists a few of the known environmental risks arising from use of GBHs.

**Table 1:** Environmental Risks

This overview of possible adverse effects associated with rising GBH use is focused on mammalian health risks. There are also many environmental and soil-ecosystem problems associated with heavy and repeated uses of GBHs affecting other organisms (for example, fish, butterflies, earthworms, beneficial soil microorganisms) [47].	ᅟ
These problems arise from the large volumes of GBHs applied across vast areas in many farming areas (for example, 80% or more of the harvested cropland in many counties in the U.S., and provinces or political jurisdictions in other countries, are sprayed with GBHs).	ᅟ
Glyphosate binds strongly to some soils, but not others. After repeated applications, it can accumulate and become a long-term source of soil and groundwater contamination [48]. The main pathways of GBH degradation are known and the principal breakdown products (AMPA, formaldehyde) could be toxic to a variety of non-target organisms. Continued long-term use of GBHs could pose a threat to soil health and fertility [47, 49], with possible adverse effects on crop productivity.	ᅟ
Low levels (50 ppb) of glyphosate have been shown to have significant negative effects on the aquatic invertebrate Daphnia magna [50]. When measured against the U.S. EPA’s Maximum Contaminant Level of 700 ppb, or the Canadian short-term (27,000 ppb) and the long-term (800 ppb) freshwater aquatic standards [51], one quickly sees how the regulatory eco-toxicological risk levels set for glyphosate are orders of magnitude too high.	ᅟ

## Section I

With respect to glyphosate-based herbicides, we are certain of the following:

### GBH Use, exposure, presence

1. GBHs are currently the most heavily applied herbicides in the world.
  Trends in the volume and intensity of GBH uses have been rising sharply since the mid-1990s, in step with global adoption of genetically engineered, glyphosate-tolerant crops [10, 52, 53]. Use of GBHs is likely to continue increasing if Roundup Ready glyphosate-tolerant maize, soybeans, cotton, canola, alfalfa and sugar beet are approved for planting in regions not now dominated by such cultivars.
2. GBHs contaminate drinking water via rainwater, surface runoff and leaching into groundwater, thereby adding drinking water, bathing, and washing water as possible routine exposure pathways [48, 54, 55].
3. The half-life of glyphosate in water and soil is longer than previously recognized. In field studies, the half-life of glyphosate in soil ranged between a few days to several months, or even a year, depending on soil composition [56]. Studies have shown that soil sorption and degradation of glyphosate exhibit great variation depending on soil physical, chemical, and biological properties. The risk of long-term, incremental buildup of glyphosate contamination in soil, surface water, and groundwater is therefore driven by highly site-specific factors, and as a result, is difficult to predict and costly to monitor.
4. Residues of glyphosate and its principle metabolite AMPA are present in nearly all soybeans harvested from fields planted with Roundup Ready soybeans [13, 16]. The intensity of glyphosate use has trended upward on most GE Roundup Ready crops. In addition, applications are now being made later in the crop cycle on GE crops. In addition, wheat, barley and other grain, and some vegetable crops are sprayed very late in the crop season to accelerate crop death, drying, and harvest operations. For these reasons, average residue levels on and in some harvested grains, oilseeds, and certain other crops are substantially higher than they were a decade ago and, as a result, human dietary exposures are rising.
5. The emergence and spread of glyphosate-resistant weeds requires farmers to spray additional herbicides, including older herbicides posing documented environmental and public health risks and/or newer, more costly herbicides to avoid crop yield losses and slow the spread of these weeds [37]. This is particularly problematic in grain and row-crop fields planted for several years with Roundup Ready GE crops. In the U.S., contending with resistant weeds has already increased total herbicide use per acre by approximately 70 % in soybeans, and 50 % in the case of cotton compared to herbicide rates on these crops in the mid-1990s when GE varieties were first introduced [10].

## Section II

We estimate with confidence that:

1. Glyphosate provokes oxidative damage in rat liver and kidneys by disrupting mitochondrial metabolism [57–59] at exposure levels currently considered safe and acceptable by regulatory agencies [4, 25, 26]. Therefore, the ADI governing exposures to GBHs is overestimated. Adverse effects impacting other endpoints are less certain, but still worrisome and indicative of the need for more in-depth research (see following sections).
2. Residues from GBHs may pose higher risks to the kidneys and liver. Metabolic studies in a variety of laboratory and farm animal species show that levels of glyphosate and AMPA in kidney and liver tissues are 10- to 100-fold (or more) higher than the levels found in fat, muscle (meat) and most other tissues^3^. Increases in the frequency of serious, chronic kidney disease have been observed among male agricultural workers in some regions in which there is a combination of heavy GBH use and ‘hard’ water [60, 61]. These possible adverse effects of GBH exposure on kidney and liver warrant a focused, international research effort.
3. There are profound gaps in estimates of worldwide human GBH exposure. Glyphosate and AMPA are not monitored in the human population in the United States, despite the 100-fold increase in use of GBHs over recent decades. In circumstances where there is substantial uncertainty in a pesticide’s dietary risk, the EPA is presumptively required by the U.S. Food Quality Protection Act (FQPA) of 1996 to impose an added safety factor of up to 10-fold in the setting of glyphosate’s cRfD. Such uncertainty can arise from gaps in the scope and quality of a pesticide’s toxicology dataset, or uncertainty in exposure assessments. Considering the uncertainties regarding both GBH safety and exposure, the EPA should impose a 10-fold safety factor on glyphosate, which would reduce the EPA chronic Population Adjusted Dose (cPAD) to 0.175 mg/kg bw/day. [Note: the U.S. EPA adopted the new term cPAD to designate a chronic Reference Dose for a pesticide that had been lowered by the Agency as a result of the application of an added, FQPA-mandated safety factor. Virtually all FQPA safety factors have reduced chronic Reference Doses by 3-fold or 10-fold].
4. Nevertheless, imposing a 10-fold decrease in glyphosate’s chronic Reference Dose, as seemingly called for in current U.S. law, should only be viewed as an interim step in the reassessment of glyphosate toxicity and risk, and re-adjustment of glyphosate uses and tolerances in food. Considerable work on glyphosate and GBH toxicity, mechanisms of action, and exposure levels must be completed before the U.S. EPA can credibly conclude that GBH uses and exposures are consistent with the FQPA’s basic safety standard, namely that there is a “reasonable certainty of no harm” from ongoing, chronic exposures to GBHs across the American population.

## Section III

Current models and data from the biological sciences predict that:

1. Glyphosate and GBHs disrupt endocrine-signaling systems in vitro, including multiple steroid hormones, which play vital roles in the biology of vertebrates [21, 22, 24, 62]. Rat maternal exposure to a sublethal dose of a GBH resulted in male offspring reproductive development impairment [21]. As an endocrine-disrupting chemical (EDC), GBH/glyphosate can alter the functioning of hormonal systems and gene expression patterns at various dosage levels. Such effects will sometimes occur at low, and likely environmentally-relevant exposures. Contemporary endocrine science has demonstrated that dose–response relationships will sometimes deviate from a linear increase in the frequency and severity of impacts expected as dose levels rise [19, 63].
2. The timing, nature, and severity of endocrine system impacts will vary depending on the levels and timing of GBH exposures, the tissues exposed, the age and health status of exposed organisms, and other biotic or abiotic stressors impacting the developmental stage and/or physiology of the exposed organism. Exposures can trigger a cascade of biological effects that may culminate many years later in chronic degenerative diseases or other health problems. Exposures leading to serious complications later in life might occur over just a few days to a month in short-lived animals, and over a few days to several months in humans.
3. The study used by the EPA to establish the current glyphosate cRfD used gavage as a system of delivery, as recommended by OECD guidelines for prenatal developmental toxicity studies, which in all likelihood underestimates both exposure and toxicity [64]. This conclusion is derived from two considerations: (i) gavage bypasses sublingual exposure, and thus overestimates the portion of the chemical subjected to first pass metabolism in the liver, and (ii) gavage stresses the experimental subjects inducing endocrine effects that can lead to artefacts including, crucially, a reduction in the difference between control and experimental groups.
4. The incidence of non-Hodgkin’s Lymphoma (NHL) has nearly doubled in the U.S. between 1975 and 2006 [65]. GBHs are implicated in heightened risk of developing NHL among human populations exposed to glyphosate occupationally, or by virtue of residence in an area routinely treated with herbicides [66]. A causal link between GBH exposures and NHL may exist, but has not been rigorously studied in human populations.
5. Uncertainty persists over the doses required to cause most of the above endocrine-system-mediated effects. Some published data indicate that doses well within the range of current human exposure may be sufficient [22, 25], whereas other studies demonstrating distinct, adverse impacts have explored high doses and exposures that are unlikely to reflect any real world levels of ingestion. Additional in vivo studies are needed at environmentally relevant doses to distinguish the combination of factors likely to give rise to endocrine-system-driven morbidity and mortality. Nevertheless, the epidemiological data described above provides evidence of heightened cancer risk in human populations at levels of exposure actually experienced in human populations.
6. Glyphosate is a chelating agent with potential to sequester essential micronutrient metals such as zinc, cobalt and manganese [67, 68]. This property of GBHs can alter the availability of these micronutrients for crops, people, wildlife, pets, and livestock. These micronutrient metals are enzymatic cofactors, so their loss has the potential to contribute to a number of deleterious effects, especially on kidney and liver function [69].

## Section IV

Existing data suggest, but do not empirically confirm, a wide range of adverse outcomes:

1. Multiple studies on GBHs have reported effects indicative of endocrine disruption [21–24]. Based on knowledge from studies of other endocrine disruptors, the developing fetus, infants, and children are most at risk. Effects following GBH exposure may not be immediately apparent, because some adverse conditions caused by early-life exposure only manifest in later stages of development and/or in adulthood. These include both acute diseases and chronic health problems. In addition, proving links between chronic disease and exposures to GBHs is made more difficult by the fact that people are routinely exposed to complex mixtures of glyphosate and other toxic chemicals.
2. The action of glyphosate as an antibiotic may alter the gastrointestinal microbiome in vertebrates [33, 70–72], which could favor the proliferation of pathogenic microbes in humans, farm animals, pets and other exposed vertebrates.
3. Increased incidence of severe birth defects in Argentina and Paraguay in areas where GE Roundup Ready crops are widely grown may be linked to the ability of GBHs to increase retinoic acid activity during fetal development [23]^4^. Glyphosate-contaminated soybean feeds used in the pork industry have also been associated with elevated rates of gastrointestinal-health problems and birth defects in young pigs [32]. Related impacts have been observed in poultry [33].
4. Some developmental studies in rats undertaken at relatively high levels of exposure suggest possible GBH-induced neurotoxicity through multiple mechanisms [73]. Replication of these studies using doses relevant to human exposures should be a high priority. Further work on GBH-induced neurotoxicity should be conducted to test whether glyphosate can act as a disruptor of neurotransmitter function given its similarity in structure to glycine and glutamate^5^.
5. GBHs may interfere with normal sexual development and reproduction in vertebrates. Experiments with zebrafish with dosing of GBH in the upper range of environmentally-relevant contamination levels, show morphological damage to ovaries [74].
6. A recent report demonstrates that environmentally relevant concentrations of commercially available GBHs alter the susceptibility of bacteria to six classes of antibiotics (for example, either raise or lower the minimum concentration needed to inhibit growth) [75]. Furthermore, GBHs can also induce multiple antibiotic-resistance phenotypes in potential human pathogens (*E. coli* and *Salmonella enterica* serovar typhimurium). Such phenotypes could both undermine antibiotic therapy and significantly increase the possibility of mutations conferring more permanent resistance traits. Since GBHs and antibiotics are widely used on farms, farm animals may be exposed to both, with a concomitant decrease in antibiotic effectiveness and increase in the diversity of newly resistant bacterial phenotypes that might find their way into the human population. Risk assessors have not previously considered the finding that herbicides might have sublethal adverse effects on bacteria, but this should be considered in future risk assessments.

## Section V

Uncertainties in current assessments persist because:

1. A steadily growing portion of global GBH use is applied in conjunction with multiple other herbicides, insecticides, and fungicides. Herbicide and other pesticide active ingredient safety levels are calculated for each active ingredient separately, despite the fact that tank mixes including two to five, or even more active ingredients account for a significant portion of the volume of pesticides applied. Regulators do not require further testing of such mixtures, nor do they conduct any additional risk assessments designed to quantify possible additive or synergistic impacts among all herbicides applied, let alone the combination of all herbicides, insecticides, fungicides, and other pesticides applied on any given field.
2. The full list of chemicals in most commercial GBHs is protected as “confidential business information,” despite the universally accepted relevance of such information to scientists hoping to conduct an accurate risk assessment of these herbicide formulations. The distinction in regulatory review and decision processes between ‘active’ and ‘inert’ ingredients has no toxicological justification, given increasing evidence that several so-called ‘inert’ adjuvants are toxic in their own right [42]. Moreover, in the case of GBHs, the adjuvants and surfactants, which include ethoxylated tallowamines, alkylpolyglycosides or petroleum distillates in most commonly used commercial formulations, alters both the environmental fate and residue levels of glyphosate and AMPA in harvested foodstuffs and animal feeds. They do so by enhancing the adhesion of glyphosate to plant surfaces, as well as facilitating the translocation of applied glyphosate from the surface of weed leaves into sub-surface plant tissues, where it exerts its herbicidal function and where rainfall can no longer dissipate the glyphosate.
3. The vast majority of GBH-toxicology studies used for regulatory assessments lack a sufficient range of dose levels to adequately assess adverse impacts that might be initiated by low, environmentally-relevant exposures^6^. Most toxicology studies examine only a high dose between the LD50 (the dose required to kill 50 % of treated animals) and the maximum tolerated dose (a dose that has high toxicity but does not kill), and then typically two lower doses (allowing for the identification of the Lowest Observed Adverse Effect Level [LOAEL] and the No Observed Adverse Effect Level [NOAEL]). Environmentally relevant doses are rarely examined [63]. A further complication arises specifically for endocrine disrupting chemicals: there are theoretical and empirical findings concluding that one cannot assume any no-impact exposure threshold for endocrine processes that are already underway because of endogenous hormones [76].
4. Residues of GBHs in plants are often present in conjunction with: (a) residues of systemic seed treatments, especially neonicotinoid insecticides (for example, clothianidin and thiamethoxam) and their adjuvants (such as organosilicone surfactants), (b) residues of systemic insecticides and fungicides applied during the season, and (c) *Bt* endotoxins in the case of GE, insect-protected *Bt* cultivars. Such mixtures and combinations are never tested, and thus it is unknown how GBHs might interact with these other agents.
5. Large-scale and sophisticated biomonitoring studies of the levels of glyphosate, its metabolites, and other components of GBH mixtures in people have not been conducted anywhere in the world. Biomonitoring studies should include measurement of glyphosate residues, metabolites, and adjuvants in blood and urine to obtain meaningful insights into internal contamination levels and the pharmacokinetics of GBHs within vertebrates^7^.
6. Adequate surveys of GBH contamination in food products have not as yet been conducted on a large scale, even in the U.S. The first and only in-depth USDA testing of glyphosate and AMPA residues in food targeted soybeans, and occurred once in 2011 [13]. Of the three hundred samples tested, 90.3 % contained glyphosate at a mean level of 1.9 ppm, while 95.7 % contained AMPA at 2.3 ppm. In contrast, the next highest residue reported by USDA in soybeans was malathion, present at 0.026 ppm in just 3.7 % of samples. Thus, the mean levels of glyphosate and AMPA in soybeans were 73-fold and 83-fold higher than malathion, respectively. Residues in animal products, sugar beet, pre-harvest treated wheat, corn silage, and alfalfa hay and sprouts are unknown, but likely much higher, given the series of recent requests by Monsanto to increase tolerance levels in a range of foods and animal feeds [12].
7. There is no thorough, up-to-date government survey of glyphosate and AMPA residues in U.S. grown Roundup Ready GE soybeans, nor manufactured foods that contain soy-based ingredients. However, changes in the rate of GBH applications on many other crops, and/or the timing of applications, have clearly increased residue levels in some circumstances. In particular, GBH uses late in the growing season as a pre-harvest desiccant have become more common. Such applications speed up the drying of crops in the field, so that harvest operations can be completed before bad weather sets in. Such harvest-aid uses are popular, especially in wet years, on wheat, canola, and other grain farms in some humid, temperate climates, such as in the UK and northern-tier states in the US. While pre-harvest uses have only modestly increased the total volume of GBHs applied, they have significantly increased the frequency and levels of residues in harvested grains, and have required GBH registrants to seek significant increases in tolerance levels. These residues are also contributing to dietary exposures via a number of grain-based products, as clearly evident in data from the U.K. Food Standard Agency’s residue testing program [14].
8. Glyphosate residues are generally uncontrolled for in the standard rations fed to animals in laboratory studies. GBH residues can often be found in common laboratory animal chows used in feeding studies, thus potentially confounding the results of GBH toxicity tests [77]. Out of 262 pesticide residues analyzed in 13 commonly used rodent laboratory diets, glyphosate was the most frequently found pesticide, with concentrations reaching 370 ppb [78]. Therefore, GBH residues should be accounted for in animal chows used in controls for GBH studies.
9. The limited data currently available on glyphosate pharmacokinetics in vertebrates are insufficient to predict transport and fate of glyphosate in different mammalian tissues, organs and fluids in the body, and to determine whether or where bioaccumulation occurs, although animal metabolism studies point strongly to the kidney and the liver.

## Section VI

The following recommendations are offered to further improve our predictive capability regarding glyphosate risks:

1. Scientists independent of the registrants should conduct regulatory tests of GBHs that include glyphosate alone, as well as GBH-product formulations. [Note: in the latest glyphosate regulatory assessment process by the German Federal Institute for Risk Assessment, the description and assessment of studies was provided by the Glyphosate Task Force, a group of 25 agrochemical companies that combined resources to jointly apply for renewal of registrations for this herbicide within Europe. By way of contrast, in order to avoid conflicts of interests, the Glyphosate Task Force was restricted to a role of observer to the evaluation of data by independent scientists at the recent WHO IARC evaluation of glyphosate’s carcinogenic potential].
2. Epidemiological studies are needed to improve knowledge at the interface of GBH uses, exposures, and human-health outcomes.
3. Biomonitoring studies examining reference populations like the U.S. CDC’s NHANES program should examine human fluids for glyphosate and its metabolites.
4. More comprehensive toxicity experiments are needed including those using “two hit” study designs, which examine early life exposures to GBHs followed by later-life exposures to chemical or other environmental stressors.
5. Because GBHs are potential endocrine disruptors, future studies should incorporate testing principles from endocrinology.
6. Future studies of laboratory animals should use designs that examine the full lifespan of the experimental animal, use multiple species and strains, examine appropriate numbers of animals, and carefully avoid contaminating GBH and other pesticides within control feeds and drinking water.
7. GBHs should be prioritized by the U.S. National Toxicology Program for safety investigations, including tests of glyphosate and common commercial formulations.

## Section VII

### Implications

1. The margin of safety between typical glyphosate and AMPA exposure levels and the maximum allowed human exposures has narrowed substantially in the last decade. The margin may well have disappeared for heavily exposed segments of the population in some countries, especially where glyphosate and AMPA are present in drinking water. In addition, farmworkers and rural residents may incur relatively high dermal absorption and/or exposures via drinking water. We conclude that existing toxicological data and risk assessments are not sufficient to infer that GBHs, as currently used, are safe.
2. GBH-product formulations are more potent, or toxic, than glyphosate alone to a wide array of non-target organisms including mammals [42, 43], aquatic insects, and fish [44]. As a result, risk assessments of GBHs that are based on studies quantifying the impacts of glyphosate alone underestimate both toxicity and exposure, and thus risk. This all-too-common shortcoming has repeatedly led regulators to set inappropriately high exposure thresholds (cRfDs, ADIs).
3. The toxicological data supporting current GBH regulatory risk assessments are out-of-date and insufficient to judge the impacts of contemporary glyphosate and AMPA exposure levels on the developing mammalian fetus, the liver and kidneys, and reproductive outcomes in humans and a variety of other animals [3, 25].
4. Most toxicological studies using advanced, modern tools and experimental designs within molecular genetics, reproductive, developmental, endocrinological, immunological and other disciplines have been undertaken in academic and research institute laboratories, and results have been published in peer-reviewed journals. Regulators have not incorporated, formally or indirectly, such research into their risk assessments. Rather, they rely on unpublished, non-peer reviewed data generated by the registrants. They have largely ignored published research because it often uses standards and procedures to assess quality that are different from those codified in regulatory agency data requirements, which largely focus on avoiding fraud [79]. Additionally, endocrine-disruption study protocols have not been codified by regulators^8^.
5. While the German Federal Institute for Risk Assessment, rapporteur for the European Food Safety Authority’s current reassessment of glyphosate, claimed to have examined more than 900 scientific studies published in peer-reviewed journals, most of the studies were deemed of limited value, and hence had little influence on the outcome of their assessment. Studies were classified of ‘limited value’ based on degree of adherence to traditional, toxicology protocols and ‘validated’ endpoints, rather than scientific rigor and relevance in understanding the mechanisms leading to adverse health outcomes. Had the German Institute used scientific quality and relevance in identifying useful studies, instead of relying on similarity to outdated methodologies and/or controversial evaluation criteria [80] (such as the Klimisch score), we are nearly certain that they would have concluded that published studies collectively provide strong evidence in support of at least a three-fold reduction in the glyphosate E.U. ADI and consequently a 15-fold reduction in the U.S. cRfD [3, 21, 25, 26].

## Conclusions

GBH use has increased approximately 100-fold since the first decade of its use in the 1970s. It is now the world’s most heavily applied herbicide. Major increases in its use resulted from widespread adoption of Roundup Ready crops that were genetically engineered to be tolerant to glyphosate. Applications of GBHs have also expanded in aquatic, estuarine, rangeland, and forest habitats.

Initial risk assessments of glyphosate assumed a limited hazard to vertebrates because its stated herbicidal mechanism of action targeted a plant enzyme not present in vertebrates. In addition, because GBHs kill nearly all actively growing plants, farmers had to apply GBHs early in the year, before crop germination or post-harvest, and so it seemed unlikely that there would be residues in harvested crops and the food supply. However, these assumptions ignored the possibility that glyphosate and its metabolites might act via other pathways, including those present in vertebrates, as well as the profound consequences of major increases in the area treated and volume applied, coupled with changes in how and when GBHs are used by farmers (e.g., on GE, herbicide-tolerant crops, and as a pre-harvest desiccant to accelerate harvest).

Evidence has accumulated over the past two decades, especially, that several vertebrate pathways are likely targets of action, including hepatorenal damage, effects on nutrient balance through glyphosate chelating action and endocrine disruption. Other early assumptions about glyphosate, for example that it is not persistent in the environment, have also been called into question, depending upon soil type. In addition, the prediction that glyphosate would never be present widely in surface water, rainfall, or groundwater has also been shown to be inaccurate.

Existing data, while not systematic, indicate GBHs and metabolites are widely present in the global soybean system and that human exposures to GBHs are clearly rising. Tolerable daily intakes for glyphosate in the U.S. and Germany are based upon outdated science.

Taken together, these conclusions all indicate that a fresh and independent examination of GBH toxicity should be undertaken, and that this re-examination be accompanied by systematic efforts by relevant agencies to monitor GBH levels in people and in the food supply, none of which are occurring today. The U.S. National Toxicology Program should prioritize a thorough toxicological assessment of the multiple pathways now identified as potentially vulnerable to GBHs. The urgency of such work was reinforced in March 2015 when the IARC concluded glyphosate is a probable human carcinogen.

We are aware of current limits on, and demands for, public funding for research. In the absence of government funds to support essential GBH research, we recommend that a system be put in place through which manufacturers of GBHs provide funds to the appropriate regulatory body as part of routine registration actions and fees. Such funds should then be transferred to appropriate government research institutes, or to an agency experienced in the award of competitive grants. In either case, funds would be made available to independent scientists to conduct the appropriate long-term (minimum 2 years) safety studies in recognized animal model systems. A thorough and modern assessment of GBH toxicity will encompass potential endocrine disruption, impacts on the gut microbiome, carcinogenicity, and multigenerational effects looking at reproductive capability and frequency of birth defects.

## Abbreviations

2,4-D: 2,4-Dichlorophenoxyacetic acid
ADI: Acceptable daily intake
AMPA: Aminomethylphosphonic acid
Bt: Bacillus thuringiensis
cPAD: Chronic Population Adjusted Dose
cRfD: Chronic reference dose
EPSPS: 5-enolpyruvylshikimate-3-phosphate synthase
EU: European Union
FQPA: US food quality protection act of 1996
GBHs: Glyphosate-based herbicides
IARC: International Agency for Research on Cancer
LOAEL: Lowest Observed Adverse Effect Level
NOAEL: No Observed Adverse Effect Level
US EPA: United States Environmental Protection Agency

## Footnotes

1 The E.U. ADI was calculated based on observed kidney (hepatorenal) effects in rat chronic toxicity studies. The “No Observable Adverse Effect Level” (NOAEL) was 31 mg/kg/day, and the “Lowest Observable Adverse Effect Level” (LOAEL) occurred at a dose of 60 mg/kg/day (determined then to be the LOAEL). A standard 100-fold safety factor was applied in converting the E.U.-set NOAEL to the ADI of 0.3 mg/kg/day. The new ADI recommended by the German regulators of 0.5 mg/kg/day is based on teratogenic effects in rabbits. The NOAEL was considered to be 50 mg/kg/day. Independent scientists argue that the 2002 determination was not based on the most sensitive species or dataset, as is required by regulatory authorities. See ref 14. Antoniou M, Habib MEM, Howard CV, Jennings RC, Leifert C, Nodari RO, Robinson CJ, Fagan J: Teratogenic effects of glyphosate-based herbicides: divergence of regulatory decisions from scientific evidence. J Environ Anal Toxicol 2012, S4:006.

2 The EPA issued an updated registration review of GBHs in 1993. Studies dating from the early 1970s through mid-1980s dominated the reference list accompanying the chapter setting forth the EPA’s estimate of GBH human health risks.

3 Table B.7.3-8 in the document “Renewal Assessment Report, Glyphosate Residue Data” (Vol. 3, Annex B.7, Dec. 18, 2013, RMS: Germany, Co-RMS-Slovakia) provides an overview of the levels of glyphosate and AMPA measured in the meat, milk, and eggs from several livestock species, as well as in the fat, meat, kidney, and livers of the animals. In most cases the levels reported in liver and kidney exceed those in other tissues by several-fold, and the levels in kidney exceed those in liver by 3-fold to over 10-fold.

4 Retinoic acid signaling plays a key role in guiding embryonic development, affecting the expression of multiple genes in a variety of cell types. Altered retinoic acid activity causes birth defects (see 58. Duester G: Retinoic acid synthesis and signaling during early organogenesis. Cell 2008, 134(6):921-931.

5 Glutamate is a common vertebrate neurotransmitter released by neurons into the synapse, and is important for learning and memory (for a review, see 59. Meldrum BS: Glutamate as a neurotransmitter in the brain: review of physiology and pathology. J Nutr 2000, 130(4S Suppl):1007s-1015s. Glyphosate’s structural similarity to glutamate creates the potential for interfering with this key signaling process.

6 “Environmentally relevant” exposures to GBHs are those that fall within the documented exposure levels arising from the way GBHs are typically used.

7 Pharmacokinetic studies project and monitor the levels of a chemical absorbed by an organism (via ingestion, inhalation, dermal absorption, or some other route of exposure), how the chemical is distributed throughout the body to specific tissues (measuring the concentrations in different organs and in the blood), how the chemical is metabolized (including which metabolites are produced, and whether the presence of these metabolites and their relative abundance is dependent on route of exposure), and finally, how a compound is excreted (e.g., in feces or urine). Pharmacokinetic studies provide a valuable link between estimates of exposure, toxicity studies, and estimates of human risk.

8 The process of establishing testing protocols for endocrine-mediated impacts has been underway in the U.S. since 1997, in response to a mandate in the 1996 Food Quality Protection Act to consider such effects in assuring a “reasonable certainty of no harm” for pregnant women, infants, and children. Seventeen years later, the EPA remains years away from codifying a new battery of tests capable of identifying the risk of low-dose, endocrine-disruption driven effects.
